# Characterization and Cytotoxic Assessment of Bis(2-hydroxy-3-carboxyphenyl)methane and Its Nickel(II) Complex

**DOI:** 10.3390/molecules29174239

**Published:** 2024-09-06

**Authors:** Ayman H. Ahmed, Ibrahim O. Althobaiti, Ebtsam K. Alenezy, Yazeed M. Asiri, Sobhy Ghalab, Omar A. Hussein

**Affiliations:** 1 Department of Chemistry, College of Science, Jouf University, Sakaka 72341, Saudi Arabia; 2 Department of Chemistry, Faculty of Science, Islamic University of Madinah, Madinah 42351, Saudi Arabia; 3 Department of Chemistry, College of Science, Taif University, P.O. Box 11099, Taif 21944, Saudi Arabia; 4 Department of Physics, College of Science, Jouf University, Sakaka 72341, Saudi Arabia; 5 Faculty of Medicine, Benha University, Benha 13518, Egypt

**Keywords:** bis(hydroxy-carboxyphenyl)methane, nickel complex, characterization, optical constants, biological impact, molecular docking

## Abstract

A condensation reaction of salicylic acid with formaldehyde in the presence of sulfuric acid led to the synthesization of the bis(2-hydroxy-3-carboxyphenyl)methane (BHCM) ligand, which was subsequently allowed to bind with nickel (II) ions. In light of the information obtained from the elemental analyses (C, H, and M), spectral (IR, MS, ^1^H-NMR, and UV–Vis) and thermal and magnetic measurements, the most likely structures of the ligand and complex have been identified. It has been suggested that the BHCM coordinates in a tetradentate manner with two Ni(II) ions to produce an octahedral binuclear complex. The SEM and TEM morphology of the compounds showed spherical shapes. An X-ray diffraction analysis indicated a considerable difference in the diffraction patterns between BHCM (crystalline) and Ni–BHCM (amorphous), and the Scherrer equation was used to calculate the crystallite size. Some optical characteristics were estimated from UV–Vis spectra. The ligand and its nickel(II) complex underlie the range of semiconductors. It was verified that for human lung (A-549) cancer, the BHCM compound displayed a significant barrier to the proliferation test in noncancerous cells (human lung fibroblasts, WI-38), which was also undertaken. To demonstrate the binding affinities of the chosen compounds (BHCM and Ni–BHCM) in the receptor protein’s active site [PDB ID: 5CAO], a molecular docking (MD) study was carried out.

## 1. Introduction

Searching for anticancer drugs is becoming more and more crucial. Every year, there is a significant rise in the incidence of cancer; around 10.9 million new cases and 67 million deaths from cancer are reported [1]. Indeed, a lack of effective therapies is causing the prevalence of cancers to rise quickly. Malignant tumors form from initial normal cells’ transformations, mutations, or genetic instability. 

Breast, colon, lung, and prostate cancers are the famous types among the majority of the population. Lung cancer begins in the lungs and is the leading cause of cancer deaths, accounting for the highest mortality rates among both women and men. The risk of developing lung cancer can be increased by workplace exposure to asbestos and other chemicals like arsenic, chromium, and nickel substances. Among the numerous consequences that lung cancer might cause are a continuously new cough accompanied by blood, difficulty breathing, headache, bone pain, loss of weight without attempting, hoarseness, and chest pain. Surgery, radiation, chemotherapy, targeted therapy, immunotherapy, and other treatments are the main approaches used to treat lung cancer cells (A549). Surgery is typically used to treat lung cancer in its early stages. However, side effects and drug resistance will come with radiation and chemotherapy; low response rates will also be a problem for immunotherapy [2]. Therefore, the devolvement of lung cancer medications and the discovery of new potent ones with fewer adverse effects is crucial.

Bis(hydroxycarboxyphenyl)methane compounds and their transition metal complexes have garnered much interest because of their fascinating characteristics. These compounds have many applications in analytical chemistry, electrochemistry, catalysis, bioinorganic and separation techniques [3,4,5,6]. Moreover, the studied bis(2-hydroxy-3-carboxyphenyl)methane (BHCM) contains two units of salicylic acid and is utilized as a poly-functional intermediate product suitable for the plastics and surface coatings industries [7]. It plays an important role in inorganic chemistry because it can easily produce chelated, colored compounds when combined with iron and copper. In addition, Mg and Ba-BHCM were prepared [8]. This ligand can behave as a multi-dentate ligand since it contains two active groups: hydroxyl and carboxyl. Of note, 5,5′-methylene disalicylic acid, which has an apparent resemblance to BHCM, has the ability to form a stable solid complex with aluminum and rare earth (M = Y, Ce, La, Sm, Gd, Nd, Ho, Pr and Yb) [9]. The physicochemical research on square planar Cu^II^, Ni^II^ and Co^II^ chelates with bis-oxime of 5,5′-methylene(salicylaldehyde) were explained by Patel et al. [10]. The thermal stability of the resulting chelates was arranged as follows: Co^II^ > Ni^II^ > Cu^II^. On the other hand, the literature data, including structural diversity, properties, pharmaceutical action on very close pamoic acid (the systematic name is 4,4′-methylenebis(3-hydroxynaphthalene-2-carboxylic acid)) and its metal pamoate complexes (including nickel(II) derivatives) have been published [11,12,13,14,15,16,17,18].

With regard to biological applications, hydroxy–carboxyphenyl compounds can be used as anti-inflammatory, antitumor, anti-atherogenic, and anticancer agents. Both hydroxycinnamic acid forms, such as caffeic and *p*-coumaric acid, and derivatives of hydroxybenzoic acid, such as protocatechuic, *p*-hydroxybenzoic, and vanillic acid, have significant anticancer action [19,20]. Catarina et al. [21] synthesized several hydroxy–carboxybenzene compounds and screened for their cytotoxic properties and potential antiproliferative in different human cancer cell lines: lymphoblastic leukemia, mammary gland and cervix adenocarcinomas. Distinct effects were found for various cell lines, which indicate a significant specificity of action of the used hydroxy–carboxybenzene compounds. Tests in noncancerous cells, human lung fibroblasts, were also undertaken to determine the toxic side effects of the studied compound [21]. Furthermore, salicylic acid, which has a synonymous structure to bis(2-hydroxy-3-carboxyphenyl)methane (BHCM), showed unquestionable proven anticancer properties [22,23]. Although certain hydroxy-carboxybenzene compounds have been shown to protect against cancerous cells, including lung cancer in humans [1,19,20,21,22,23], bis(2-hydroxy-3-carboxyphenyl)methane (BHCM) has not yet been the subject of any research.

This work was designed to explore the optical, biological, and crystalline properties of bis(2-hydroxy-3-carboxyphenyl)methane (BHCM) and its corresponding Ni(II) complex, in addition to their molecular structure. These properties could be employed in multiple applications such as chemistry, medicine, materials/crystals engineering, semiconductors, and optoelectronic devices, including photo-detectors, solar cells, etc. For this purpose, several techniques have been employed. The optical parameters like band gap, refractive index, optical conductivity, and penetration depth (W) were determined. In order to demonstrate the ligand’s potential use in pharmacology, the work attempts to show the inhibitory impact of the free ligand (BHCM) on human lung cancer cells and human lung fibroblast normal cells. Also, molecular docking simulations of BHCM/Ni(II) –BHCM and the receptor protein (5CAO) implicated in the etiology of human lung malignancy were performed to demonstrate the binding affinity of the free ligand and its associated complex with its potential target.

## 2. Results and Discussion

**Scheme 1:**
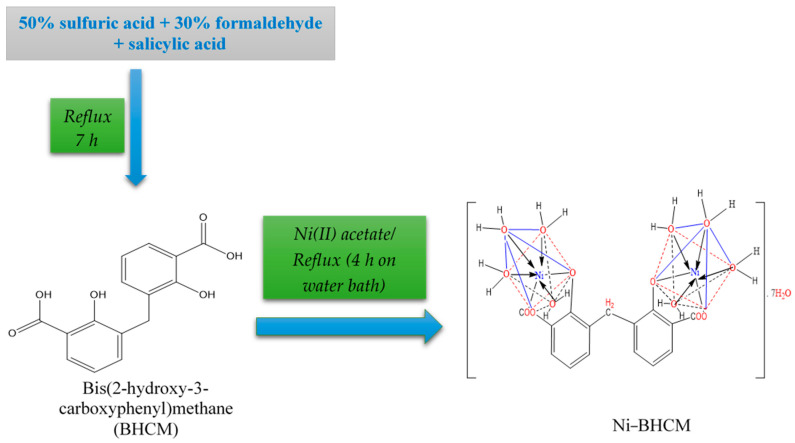
Diagram illustrating the Ni–BHCM creation.

Throughout the elemental analyses (C, H, and M), spectral (IR, MS, ^1^H-NMR, and UV-Vis), thermal (TG) and magnetic measurements, the structural formula of the generated molecules (BHCM and Ni–BHCM) have been identified. Scheme 1 presents the formation and most plausible structures of the BHCM ligand and its associated Ni(II) complex based on the mentioned conclusions in Section 3.

### 2.1. Morphology (SEM and TEM)

**Figure 1:**
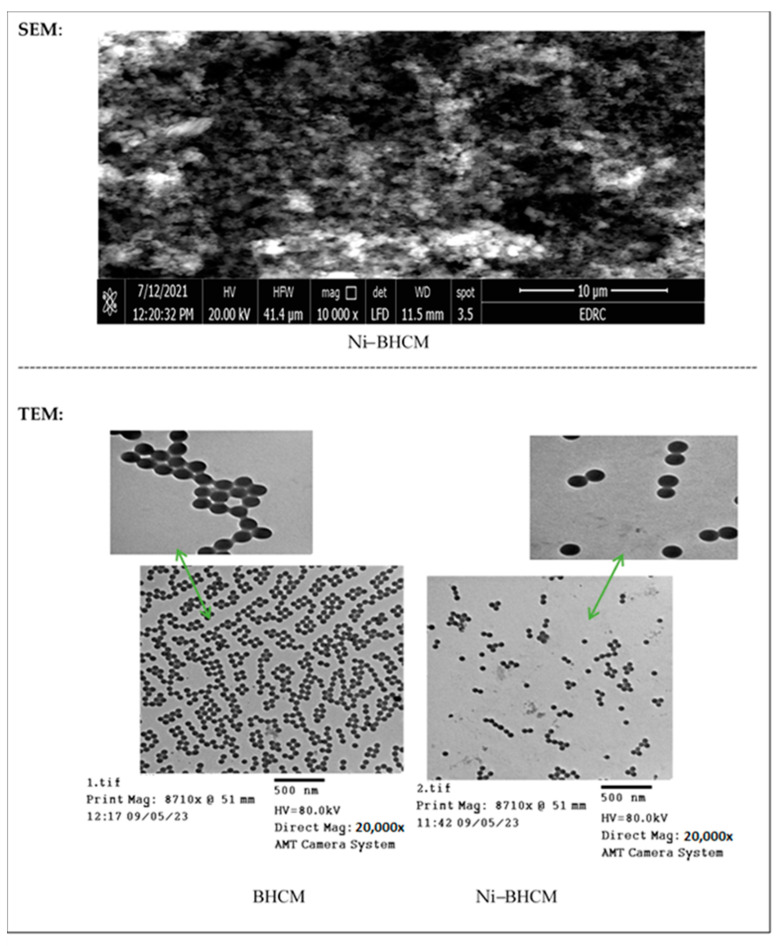
BHCM and Ni–BHCM morphological pictures.

The surface shape of the nickel complex was imaged by a scanning electron microscope (SEM), and Figure 1 demonstrates the resulting SEM micrograph. The complex revealed a spherical, granule-like structure. To verify the morphological characteristics, a transmission electron microscopy (TEM) investigation was also performed; Figure 1 displays the TEM pictures of BHCM and Ni–BHCM at the same magnification. Both ligand and complex micrographs showed regular spherical shapes, and the particles of samples seemed heterogeneous with a single regular form [24]. According to the TEM images, the average particle sizes of BHCM and Ni–BHCM are 150 and 116 nm, respectively. Obviously, the Ni(II) complex SEM findings match the TEM data well.

### 2.2. Powder XRD 

**Figure 2:**
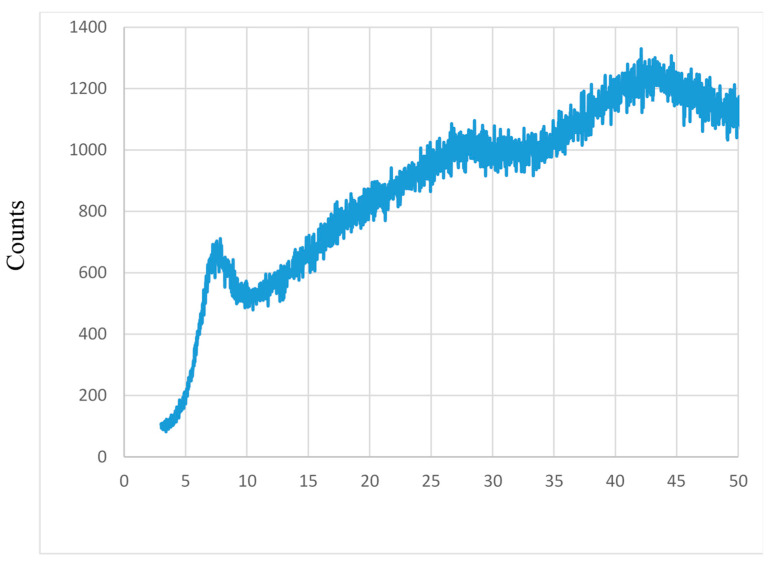
XRD graph of Ni–BHCM.

The X-ray diffraction patterns of BHCM (Figure S4) and Ni(II) complex (Figure 2) were recorded. Table S2 gives the crystallite size and inter-planar distances d (Å) from the diffraction data. The collected data indicated that the BHCM is crystalline, and its constituent molecules are arranged in a single geometric form. The sharp peaks in the XRD patterns actually prove the crystallinity of the BHCM [25]. For Ni–BHCM, the acquired amorphous nature suggested that the constituent particles are randomly grouped. The successful chelation process can be verified by the notable dissimilarity between the ligand’s and Ni–BHCM’s diffraction patterns. It is important to note that the Ni(II) Schiff base complex [{Ni(L)(H_2_O)Cl} where HL = 2-((pyridin-3-ylmethylene)amino)phenol], was produced using the same reflux procedure as this work, and the resulting particles had an amorphous structure. It has been reported that the amorphous state production is caused by the polymorphism phenomenon, which is found for the Ni(II) complexes and suggests the existence of various conformers [26]. On the other hand, the PXRD experiment was carried out for {[Ni(PA).(bpp).(H_2_O)_2_].DMF}n and [Ni_1/2_(pam)_1/2_.(py)_2_.(H_2_O)_2_]_n_, (H_2_PA—pamoic acid, bpp = 1,3-bi(4-pyridyl)propane, py—pyridine). The simulated patterns (generated from single-crystal diffraction data) and the experimental patterns’ diffraction peaks closely matched, according to PXRD studies, demonstrating the compounds’ high phase purity [17,18]. To ascertain the samples’ crystallite sizes, the Debye–Scherrer equation [27,28] was employed; it is not applicable in limits larger than 200 nm. D = kλ/βcosθ is the Scherrer equation, where D is the average crystallite size (Å), λ is the X-ray radiation wavelength (Cu K_α_ = 1.54 Å), k is the constant, assumed to be 0.94, The diffraction angle, θ, is measured in degrees, and β is the line broadening at full width at half-maximum height (FWHM) in radians. It has been found that the diameter of the BHCM crystallite was 14.4. Ni–BHCM’s amorphous state made it impossible to use the Debye–Scherrer equation.

### 2.3. Optical Properties

The optical parameters like band gap, refractive index, optical conductivity, and penetration depth (W) of the nickel complex were estimated and compared with its related BHCM ligand (please see Supplementary Materials, Figure S5).

**Figure 3:**
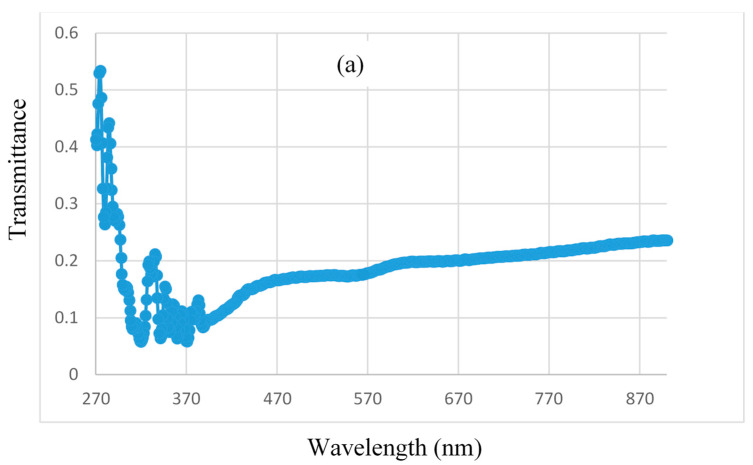

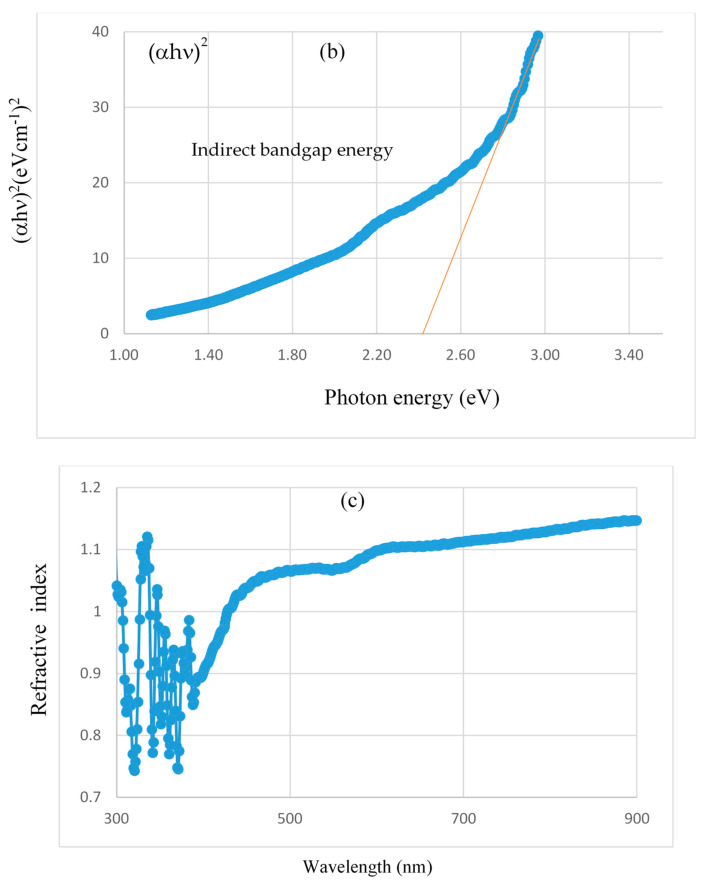

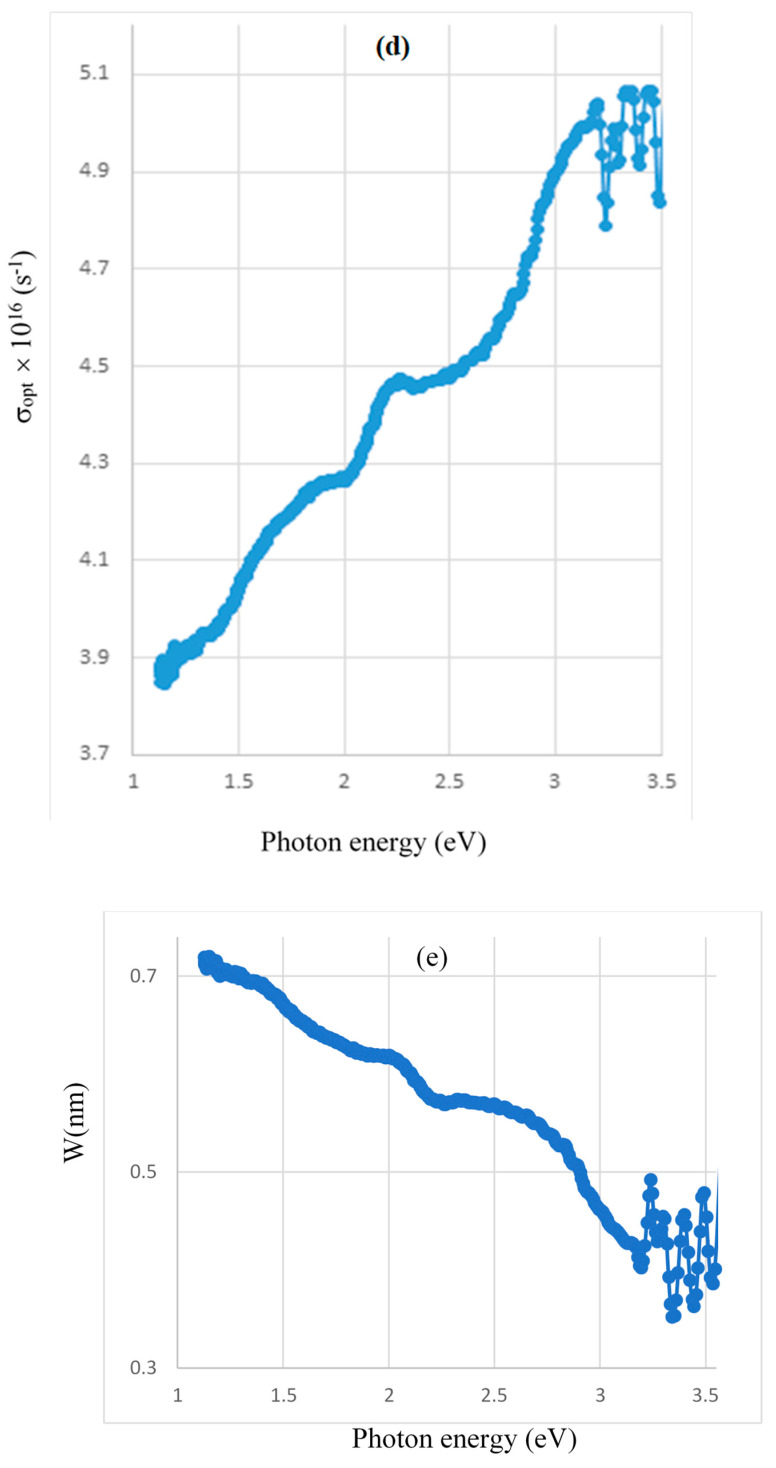
Changes in the nickel complex’s optical parameters include (**a**) transmittance—T, (**b**) band gap energy—E_g_, (**c**) refractive index—n, (**d**) optical conductivity—σ_opt_ and (**e**) penetration depth—W.

**Figure 4:**
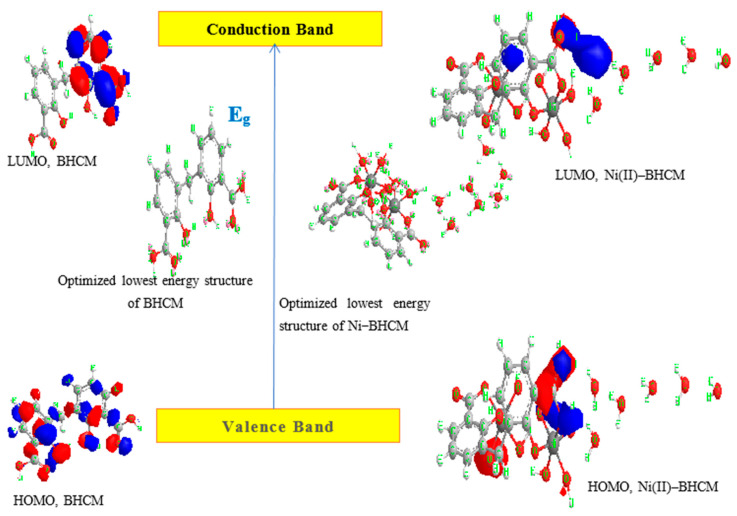
HOMO and LUMO transition, energy and optimized lowest energy structure of the ligand and nickel(II) complex. BHCM: [MM2 Minimization: Stretch: 2.1471, Bend: 8.8211, Stretch-Bend: −0.1542, Torsion: −16.3849, Non-1,4 VDW: −9.7095, 1,4 VDW: 12.6725, Dipole/Dipole: 0.3568, Total Energy: −2.2511 kcal/mol] and [MMFF94 Minimization: Final Energy: 36.2527 kcal/mol]. Ni–BHCM: [MM2 Minimization: Stretch: 118.3621, Bend: 331.6871, Stretch-Bend: −0.2150, Torsion: −9.8166, Non-1,4 VDW: −58.7081, 1,4 VDW: 4.6860, Dipole/Dipole: −62.5274, Total Energy: 323.4682 kcal/mol] and [MMFF94 Minimization: Final Energy: 683.54 kcal/mol].

The transmittance spectra of the complex have been shown in Figure 3a and utilized to study the optical properties. The term “energy gap” (E_g_) describes the energy needed for an electron to transition from the valence band to the conduction band. This transition allows the electron in the conduction band and the corresponding electron hole in the valence band to move freely within the crystal lattice, facilitating electrical conductivity. HOMO and LUMO are the acronyms for “highest occupied molecular orbital” and “lowest unoccupied molecular orbital,” respectively, in the field of chemistry. Electrical current can flow when electrons shift from the largely filled valence band to the mostly empty conduction band. Thus, one of the main determinants of the solids’ electrical conductivity is their band gap. Because the valence and conduction bands overlap, substances with large band gaps (>4 electron volts, or eV) are often insulators, semiconductors have modest band gaps (0.1 up to ~4 eV), and conductors have very small or no band gaps. Due to the dependence of the optical properties (the ability to absorb light) on the band gap values, the band gap energy of the prepared complex has been quantified. It is evident in Figure 3b and Figure S5b that the indirect optical band gap values, E_g_, of BHCM and Ni–BHCM, are 3.23 and 2.42 eV, respectively. The E_g_ values of Ni(II) complexes derived from (3E)-3-aza-5-(hydroxyimino)-1,4-diphenylpent-3-en1-ol mono hydrate and oligo-2-[(4-chlorophenyl)iminomethylene]phenol ligands were reported to be 2.32 and 3.85 eV, which may behave like semiconducting materials [29,30]. According to the findings, the ligand’s conduction resembles that of ZnO (E_g_ = 3.37), but that of Ni–BHCM is more akin to GaP (E_g_ = 2.26) and GaS (E_g_ = 2.5) [31]. Subsequent clarification explains why the E_g_ value of BHCM is greater than that of the corresponding Ni(II) complex. As reported [32], it might be viewed that after chelation, the band gap is less due to an increase in the width of the localized levels, which facilitates the movement of electrons from HOMO to LUMO energy state. This indicates that the electric conductivity of the Ni–BHCM complex is greater than free BHCM. The displayed constructions in Figure 4, determined using ChemDraw Professional 16, refer to the most stable and comfortable shapes. A refractive index is a crucial characteristic for optical applications that determines the speed at which light moves through the investigated compounds. The slower the refractive index, the higher the light travels, which causes a correspondingly decreased change in the direction of the light within the compound. For use in optic devices, including switches, modulators and filters, the study of optical refractive indices is important. In addition, the refractive index is a significant physical parameter that is frequently employed in chemistry to determine purity. For the above reasons, it is important to detect the refractive index for the ligand and complex. The compounds’ refractive index values (n) may be approximately expressed by the relationship [33,34]:

$$n=\frac{1+\sqrt{R}}{1-\sqrt{R}}$$

where R is the normal reflectance that has been estimated by using the relation R + T + A = 1. Figure 3c displays the variations in refractive index (n) with respect to wavelength (λ, nm). It is noted that variations in wavelength of the incident light beam cause changes in the refractive index because some interactions take place between photons and electrons [35]. Furthermore, this figure demonstrates how complexation influences the free ligand’s refractive index, where there is a difference in its n values by chelation. From the data shown in Figure 3c and Figure S5c, it clearly appears that the refractive index rises rapidly (ligand: 1→1.2 and complex: 0.89→1.05), after which it stays almost constant. Indeed, Karipcin et al. demonstrated the influence of the refractive index on wavelength for the dioxime ligand and its copper complex [31].

Depending on the wavelength/frequency of the incident light and refractive index values (n), the optical conductivity (σ_opt_) is given as [36]:

σ_opt_ = nkν = nck/λ

where n, c, k, ν, and λ are the refractive index, velocity of the light, extinction coefficient (αλ/4π), frequency and wavelength, respectively. The variation of σ_opt_ as a function of photon energy (hv) is shown in Figure 3d and Figure S5d. The figure clearly illustrates how, for two compounds, the σ_opt_ value changes and rises with increasing photon energy. It is evident that when compared to BHCM, the Ni–BHCM complex has a greater optical conductivity. It is noteworthy to add that σ_opt_ change as a function of hv for Zn-incorporated TiO_2_ was recorded by Paul et al. It was carefully observed that as photon energy increases, the σ_opt_ value rises [36]. 

The penetration depth of a substance determines the extent of light that can pass through it. This depth, sometimes referred to as skin depth, is attained when the internal radiation intensity of the material falls to around 37% of its initial level. Penetration depth of light (W) through two compounds has been calculated using the relation:

W = λ/4πk

where k and λ are the extinction coefficient and wavelength, respectively. Therefore, electromagnetic radiation may penetrate a material very deeply or immediately go away, depending on the wavelength of the radiation and the properties of the material. In fact, depth can provide greater significance and insight to the content. Figure 3e and Figure S5e display the fluctuation of W vs. photon energy. Noticeably, the free ligand exhibits greater penetration than the Ni–BHCM complex up to photon energy equivalent to 3.55 eV.

### 2.4. Cytotoxic Impact

#### 2.4.1. Against Human Lung Cancer Cells (A-549)

**Figure 5:**
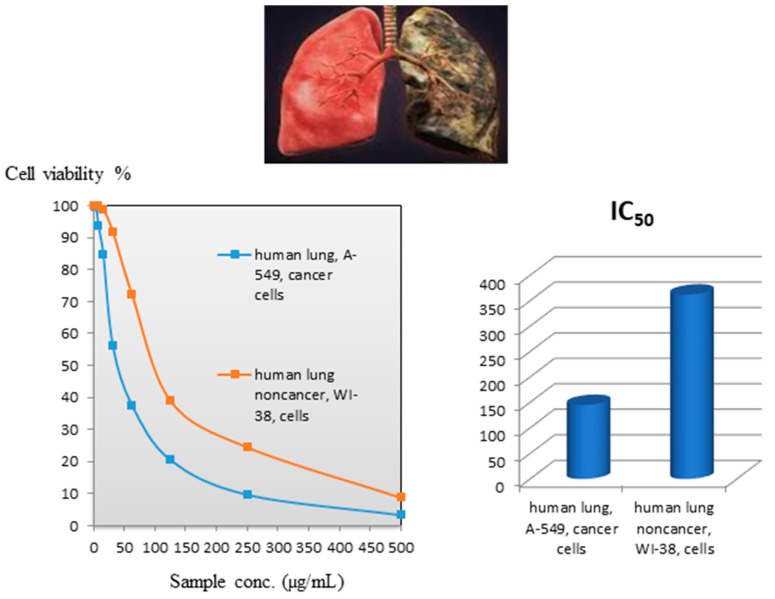
IC_50_ and assessment of BHCM’s cytotoxicity on the human lung carcinoma (A-549) and fibroblast normal cells (WI-38).

The BHCM ligand was evaluated against lung cancer cells in humans, A-549. However, Ni–BHCM was not able to be tested in vitro due to the complex’s insolubility in solvents, which limited the evaluation of its cytotoxic potential. Figure 5 displays the tumor shape and cell viability values. The ligand concentration that inhibits half of cell multiplication (IC_50_) was determined to be 144.8 ± 10.8 µM for lung cancer. The impact of BHCM inhibition can be illustrated based on a variety of factors that could contribute to the inhibition of BHCM, including (1) reaction of BHCM ligand with metal ions of the cell fluid may affect cell functions and stop their activity, (2) ligand–protein interactions inside the active site, such as covalent, van der Waals, hydrophobic (staking), and H-bonding, and (3) BHCM can bind to the metal at the active site either by displacing water or by use of a vacant site. This interaction leads to the formation of stable chelates with the transition metals found in cancer cells, which prevents a number of crucial enzymatic activities from happening [37,38]. This action caused the enzymes to be destroyed and, thus, the death of the cancerous cells. The BHCM action can really be considered a characteristic result, especially as it closely resembles salicylic acid, which is used in many pharmaceutical formulations as an anti-inflammatory and analgesic and is often included in aspirin, which is used to control heart disease. In addition, salicylic acid is present in foods, particularly fruits and vegetables, and is used as ointments, creams, foam, or soap (cosmetic additives) to treat some skin problems [39,40].

#### 2.4.2. Against Human Lung Noncancerous Cells 

BHCM was assessed towards the normal human lung fibroblast cells, WI-38, to obtain an indication of the cytotoxicity of BHCM in normal cells. Figure 5 exhibits the cell viability values. A concentration of 361.9 ± 17.8 µM was determined to be the critical concentration (CC_50_) required to kill 50% of healthy cells. The above value, even under proliferation conditions, was ~2.5 times that found in human lung carcinoma (A-549). This finding suggests that the compound does not significantly affect the proliferating normal cells [40]. Undoubtedly, this outcome was conjectured since BHCM closely resembles salicylic acid, a non-toxic chemical that occurs naturally [39,40].

### 2.5. Molecular Docking Simulation

**Figure 6:**
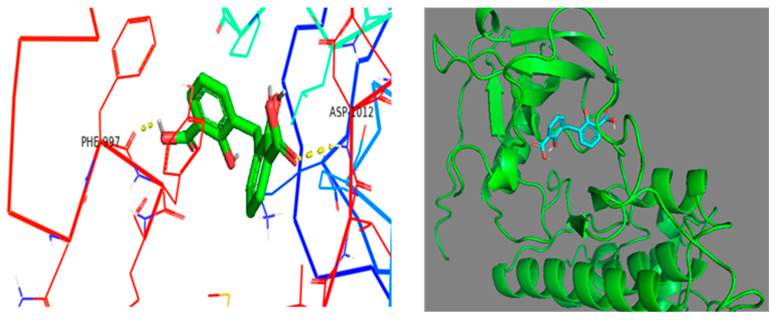
Interaction of BHCM with 5CAO as a receptor.

**Figure 7:**
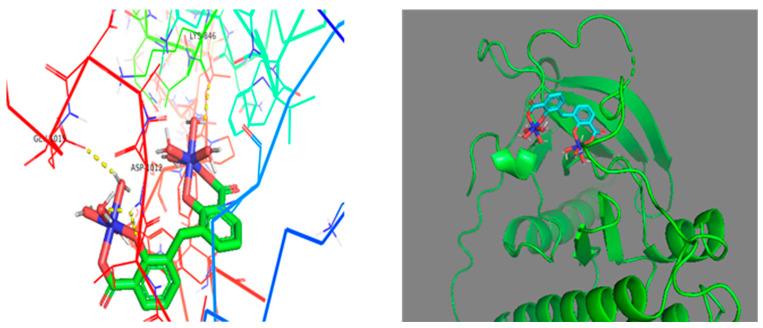
Interaction of the Ni–BHCM complex with 5CAO as a receptor.

Since the double-mutated EGFR is one of the main oncogenic proteins implicated in the etiology of a large fraction of lung cancer cases, this protein was chosen for molecular docking in order to find molecules capable of binding to the protein [41,42]. Table 1 and Figure 6 and Figure 7 depict the hydrogen bonding interactions between chemical compounds and the protein’s amino acid residues, as well as the binding affinity/docking score, which indicates the strength of binding with the receptor. In general, molecules and proteins are thought to have good docking activity if their docking score is greater than 5, and if their docking result is greater than 7, they are said to have strong docking activity [43]. PyMol software 2.5.8 (Python 3.9.18 64bit) shows that BHCM forms H-bond interactions with Phe-997 (via the OH of the first carboxyl group) and Asp-1012 (through the C=O of the second carboxyl group) of the 5CAO protein, with a binding affinity of −7.8 kcal/mol (Figure 6). Ni(II)–BHCM bonded to three amino acids of the protein, Glu-1015, Lys-846 (via an H-bond with the H of coordinated water), and Asp-1014 (via a bridged H-bond with the O of coordinated water and O of coordinated ligand hydroxyl), with an enhanced/greater binding affinity of −10.3 kcal/mol (Figure 7). Interestingly, the virtual screening showed that the selected compounds, particularly the nickel complex, are potent inhibitors of the target proteins.

## 3. Experimental

### 3.1. Materials and Techniques

All BDH reagents were utilized without any further purification. The fetal bovine serum, DMEM, HEPES buffer solution, L-glutamine, gentamicin, and 0.25% Trypsin-EDTA were provided by Lonza (Verviers, Belgium). Trypan Blue dye, MTT, and dimethyl sulfoxide (DMSO) were sourced from Sigma-Aldrich in St. Louis, MO, USA. The mammalian cell lines A-549 (human lung cancer) and WI-38 (normal human lung fibroblast) were obtained from the American Type Culture Collection (ATCC, Rockville, MD, USA). Using a Griffin & George melting point device, the melting point was measured in an open capillary tube and is uncorrected. Using a Perkin Elmer 2400 (Perkin-Elmer company, Überlingen, Germany), the element analysis for carbon (C) and hydrogen (H) was conducted. An atomic absorption spectrometer, model number GBC932/933, was used to estimate the metal concentrations (wt%). For the Thermo Scientific GC-MS type ISQ (Thermo Scientific company, Göteborg, Sweden), The mass analyzer’s direct inlet was used to perform the MS spectrum. The electron impact mode was used for the mass spectrometry. Thermo Scientific iS50 FT-IR spectrometer (Thermo Scientific company, Göteborg, Sweden) was used to record the IR spectra using the ATR method. At room temperature, ^1^H-NMR (400 MHz) measurement in dimethylsulfoxide (DMSO-d6) was performed using a Bruker spectrometer (Bruker company, Bremen, Germany). Tetramethylsilane, or TMS, is used to express chemical shifts in parts per million, or ppm. With a Perkin-Elmer Lambda 35 UV–Vis Spectrophotometer (Perkin-Elmer company, Überlingen, Germany), electronic spectra were recorded in the 200–1100 nm range using the Nujol–Mull method. Using the magnetic susceptibility balance of the Johnson Metthy and Sherwood models at room temperature, the mass susceptibility was measured and the effective magnetic moments µ_eff_ were computed [44,45]. Thermal analysis under nitrogen was executed with a Shimadzu 50 H thermal analyzer (Shimadzu company, Carlsbad, CA, USA). The crystallinity of BHCM and Ni–BHCM complex was investigated using Cu target K radiation (1.54 Å, 40 kV, and 40 mA) at two angles ranging from 3 to 80 degrees using Germany’s Bruker Co. D_8_ Discover XRD instrument (Bruker company, Bremen, Germany). 

Electron micrographs were taken at 80 kV using a JEOL GEM-1010 transmission electron microscope at the Regional Center for Mycology and Biotechnology (RCMB) at Al-Azhar University (Egypt). At the Egyptian Desalination Research Center (EDRC) within the Desert Research Center (DRC) in Egypt, surface images of the nickel complex were captured using a Quanta FEG 250 scanning electron microscope (manufactured by FEI Company, Hillsboro, OR, USA). The samples were placed on SEM stubs for analysis. The scanning electron microscopy (SEM) was conducted under conditions of a 10.1 mm working distance and a 20 kV excitation voltage for the in-lens detector. For transmission electron microscopy (TEM) analysis, a drop of the solution was placed on carbon-coated copper grids (CCG) and allowed to dry at room temperature to facilitate water evaporation. Electron micrographs were taken at 80 kV using a JEOL GEM-1010 (JEOL company, Welwyn Garden City, UK)provided by the Regional Center for Mycology and Biotechnology (RCMB) at Al-Azhar University in Egypt. The optical band gap energy (E_g_) of the BHCM and its associated Ni(II) complex was calculated to assess the conductivity of the isolated compounds [46,47,48]. The absorption coefficient (α) was estimated using the equation α = 1/d ln(1/T), where d represents the cell width and T denotes the measured transmittance. The optical band gap was determined using Tauc’s equation, αhυ = A (hυ − Eg)^m^, where m equals 1/2 for direct transitions and 2 for indirect transitions, with A being a constant independent of energy. The α values derived from the first equation were used to create a plot of (αhυ)^2^ against hυ, from which the linear segment of the curve was extrapolated to (αhυ)^2^ = 0 to ascertain the indirect band gap. The HOMO and LUMO shapes, total energy (molecular mechanics, MM2), final energy (molecular mechanics forcefield, MMFF94) and optimized lowest energy structure of ligand and nickel (II) complex were determined using ChemDraw Professional 16. Specific settings used in Chem3D for energy minimization are as follows: (1) MM2 minimization (minimum RMS gradient = 0.010, step interval = 2, frame interval = 10, target temperature = 300 K; parameter quality: all parameters used are finalized; job type: minimize energy to minimum RMS gradient of 0.010 display every iteration). (2) MMFF94 minimization (preferences: enable multiprocessor support, maximum number of iterations = 500, minimum RMS gradient = 0.100; electrostatic: dielectric constant = 1, dielectric exponent = 1; van der Waals calculation: shift function, cut off parameter = 10). A cytotoxicity screening was conducted at RCMB, Al-Azhar University (Egypt).

### 3.2. Syntheses (BHCM and Ni–BHCM) 

BHCM ligand: bis(2-Hydroxy-3-carboxyphenyl)methane (Scheme 1) was isolated as follows: For seven hours, 180 g of 50% sulfuric acid, 9.4 g of 30% formaldehyde and 27.6 g of salicylic acid were refluxed. After cooling and filtering out the separated powder, a hot water/ethanol combination solution was used to gently clean it in order to get rid of any remaining unreacted compounds. Following the extraction and recrystallization of the powder from the acetone, it was left to air dry for 48 h [Lit./Exp. m.p = 238 °C] [7]. While insoluble in water, the BHCM is soluble in normal ether, alcohol, acetone, dimethylformamide (DMF), carbon tetrachloride, and dimethylsulfoxide (DMSO). Off-white powder, Yield 90%, M.p. = 238 °C; Exp/Lit. Found (%): C, 62.60; H, 4.04 C_15_H_12_O_6_. Calculated (%): C, 62.50; H, 4.20. Mass spectrometry (MS), Figure S1. M^+^: Found (m/e): 288.35. C_15_H_12_O_6_. Calculated (m/e): 288.35. ^1^H NMR (400 MHz, DMSO-d_6_), Figure S2, δ_ppm_: 3.8 (CH_2_, d), 6.7–7.9 (aromatic protons, m), 11.2 (COOH, s, broad) and 11.6 (phenolic OH, s, broad). The chemical shift was observed in water and solvent (DMSO) at approximately 3.8 and 2.5 ppm, respectively [49]. The vanishing of -COOH and -OH signals with the injection of D_2_O indicated the proper positions of the -COOH and -OH groups. These two signals disappeared in D_2_O since they were attributed to exchangeable protons. Broadening of the COOH and phenolic OH signals supported the formation of intermolecular hydrogen bonding for these groups, which is in line with IR observations. IR, Figure 2, Table S2, (ATR, cm^−1^): 3150 (νOH_phenolic_), 1281 (νC-O_phenolic_), 1200 (δOH_phenolic_), and 1648 (νC = O_carboxylic_) [50,51], 1609, 1585, 1501 and 1435 cm^−1^ (aromatic C=C), 2905 (υCH_asym,_ methylene), 2836 (υCH_sym,_ methylene) and 754 (δCH_rocking,_ methylene), ~3033 (υCH_aromatic_) and 791 (υCH_aromatic_ out of plane deformation). The presence of intermolecular hydrogen bonding (O–H….O-H) between BHCM molecules in the solid state is indicated by the detection of phenolic OH at a lower frequency (3150 cm^−1^). This intermolecular hydrogen bond is suggested to be responsible for the broadening of the υOH_phenolic_ peak, while a peak resulting from an intramolecular hydrogen bond appears sharp. A significant decrease in the frequency of the υOH_phenolic_ band reflects the strength of this interaction. Additionally, the existence of an intermolecular hydrogen bond between the COOH groups of BHCM molecules is supported by a broad band observed in the 2395–2650 cm^−1^ range, indicative of a dimer structure. The (C=O) stretching frequency for the carboxyl group is lower than the typical range (1700–1730 cm^−1^) due to internal conjugation (where the lone pair on the carboxyl OH resonates with C=O) and the dimeric nature of BHCM. UV-Vis spectroscopy (Figure S3, Nujol), the maximum wavelengths (λ_max_ in nm) are noted as follows: 361(n→π*, carboxylic C=O), 351 (n→π*, phenolic OH), 341 (π→π*, carboxylic C=O), 319 (π→π*, phenolic OH) and 280 (π→π*, phenyl).

**Figure 8:**
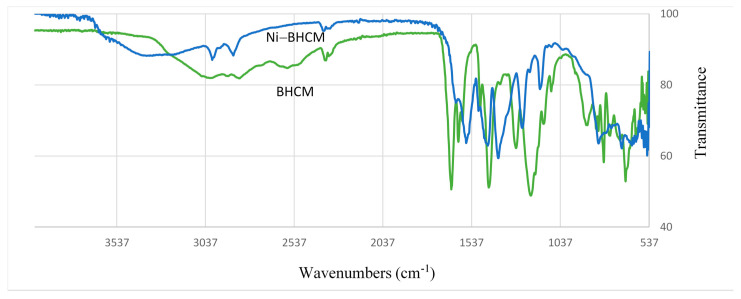
FT-IR spectrum of Ni–BHCM complex.

Ni–BHCM complex: The solid compound was produced by mixing ethanolic solutions of 0.06 mole Ni(II) acetate and 0.03 mole BHCM. In a water bath, the reaction mixture was refluxed for four hours. Before being air-dried, the colored granules were filtered and extensively washed with hot ethanol to eliminate the unreacted ligand. The complex is insoluble in water, normal ether, alcohols, acetone, carbon tetrachloride, ethyl acetate, DMF and DMSO. The insoluble nature of the Ni (II) complex in organic polar solvents like ethanol, acetone, DMF, and DMSO suggests that the polar groups (OH, COOH) of BHCM have been deprotonated and coordinated to the nickel center. This might be seen as evidence that the deprotonated hydroxyl and carboxyl groups were capable of coordinating with the central nickel metal. Light cocoa, yield 88%, M.p. > 300 °C. Found (%): C, 26.70; H, 5.60; Ni, 17.05. C_15_H_38_O_21_Ni_2_ Calculated (%): C, 26.82; H, 5.70; Ni: 17.47. IR, Figure 8, Table S1, (ATR, cm^−1^): 1245 (νC-O_phenolic_) [50,51], 1560 (νC=O_carboxylate_), 544/551(νNi–O, phenolate/carboxylate), 3425, 8760 and 610 (νOH, δOH_rocking_ and δOH_wagging_ respectively, coordinated water). A comparison of the IR spectrum of BHCM with this of the complex (Figure 8, Table S1) suggested that BHCM acts as a tetradentate ligand that coordinates to 2Ni^2+^ ions through the deprotonated (-COOH) and phenolic (-OH) groups. The formation of the Ni–BHCM complex is indicated by (1) a negative shift in the υ(C–O) band from BHCM (1281 cm^−1^) to Ni–BHCM (1245 cm^−1^); (2) the disappearance of the (C=O) band of the carboxyl group (L: 1647 cm^−1^, Table S1), replaced by the (C=O) band of the carboxylate in the complex at 1560 cm^−1^; and (3) the absence of δ and υ(OH)phenolic bands in the ligand spectrum following chelation. (4) More proof is provided by the persistence of two new bands in the 530–560 cm^−1^ range, centered at 544/551 cm^−1^. These bands are most likely caused by the Ni–O vibrations of phenolate and carboxylate [50,51]. The stretching broadband at 3425 cm^−1^ and two weaker bands corresponding to the wagging (OH_water_: 610 cm^−1^) and rocking (OH_water_: 8760 cm^−1^) vibrations in the complex are indicators of coordinated water.

**Figure 9:**
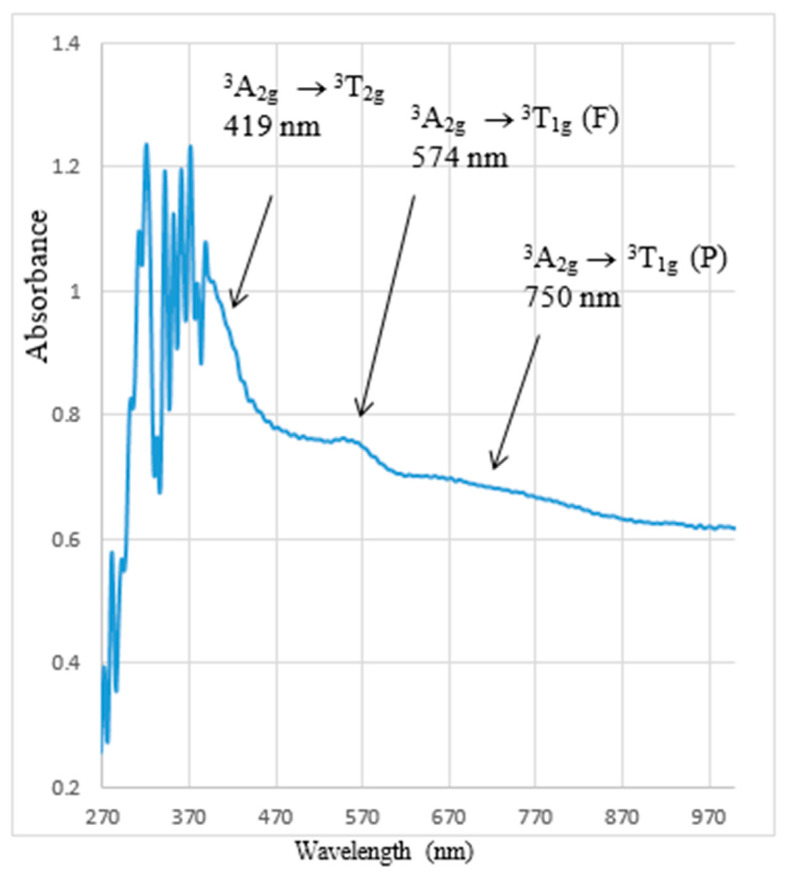
Electronic spectrum of Ni–BHCM.

**Figure 10:**
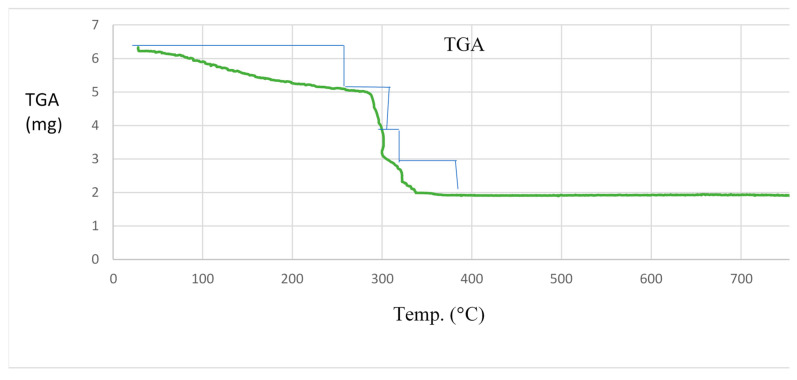
TGA of Ni–BHCM.

UV–Vis, Figure 9, Nujol, λ_max_ (nm): Identical BHCM peaks, 419 (^3^A_2g_ → ^3^T_1g_(P)), 560 (^3^A_2g_ → ^3^T_1g_(F)) and 680 (^3^A_2g_ → ^3^T_2g_) suggesting an octahedral arrangement (Oh symmetry) surrounding nickel(II) ions [52]. Of note, the reported octahedral [Ni(bipy)_3_]^2+,^ which contains the bidentate bipy-ligand (like bidentated BHCM), supports this claim. It exhibited two bands at 520 and 790 nm, which are designated as ^3^A_2g_ → ^3^T_1g_(F) and ^3^A_2g_ → ^3^T_2g_ [52]. Furthermore, metal–organic frameworks, [Ni(pamoic acid)(1,3-bi(4-pyridyl)propane)(HO)_2_].DMF}_n_ has been solvothermally synthesized, and the coordination geometry around the Ni(II) ion was described as a slightly distorted octahedron [17]. Effective magnetic moment (μ_eff_, BM): 5.35. The value is almost twice as large as that of the magnetic moment calculated by the spin-only formula (μ_s_ = 2.83 BM for two unpaired electrons). The justification for this finding is that two closed Ni(II) ions are present in the molecular structure (Scheme 1), metal–metal interaction (Ni^II^↑↑ … ↑↑Ni^II^), which results in a magnetic reinforcement and a rise in magnetism [53]. Thus, the value of 4.9 B.M. for binuclear nickel(II) complex in octahedral geometry may be due to a very strong ferromagnetic coupling between two high spin Ni(II) centers. This claim can be elucidated as follows: (1) The BHCM ligand is more flexible due to the presence of two phenyl rings joined by a twisted -CH_2_- moiety. This gives the two phenyl rings ample freedom to twist around the –CH_2–_ [15], resulting in close proximity for the two Ni(II) centers. (2) An orbital contribution is present. It is conceivable to transform the t_2g_ orbitals into each other by rotating 90° [54]. (3) The nickel centers’ approach is not impeded by small and planarity-coordinated water molecules. Thermal analysis (TG), Figure 10, Found (%): 7H_2_O_crystalline_, 19.1 (40–247 °C); 8H_2_O_coordinated_, 20.0 (250–3001 °C). Calculated (%): 7H_2_O_crystalline_, 18.8 (40–247 °C)*;* 8H_2_O_coordinated_, 21.4 (250–3001 °C). Two more weight loss peaks (301–375 °C) result from the Ni(II) complex decomposition and producing metal oxides in the end. The results show that the generated complex is thermally stable up to 250 °C and consistent with the specified configuration in Scheme 1.

### 3.3. Cell Viability Assay 

#### 3.3.1. Human Lung Carcinoma Cells (A-549) 

To support cell growth, gentamicin (50 µg/mL) and 10% inactivated fetal calf serum were added to the RPMI-1640 medium. The cells were subcultured two to three times a week and maintained at 37 °C in a humidified atmosphere with 5% carbon dioxide.

For the antitumor assays, tumor cell lines were suspended in the medium at a concentration of 5 × 10^4^ cells/well cells per well in Corning 96-well tissue culture plates [55,56]. The plates were then incubated for 24 h. Following this, the analyzed BHCM was added to three duplicates of the 96-well plates to create ten different concentrations. Six vehicle controls, consisting of medium or 0.5% dimethysulfoxide, were included for each 96-well plate. After a 24-h incubation, the number of viable cells was assessed using the MTT assay. The medium in the 96-well plate was promptly replaced with 100 µL of fresh, phenol red-free RPMI 1640 culture medium. Afterwards, 10 µL of a stock solution containing 12 mM MTT was introduced into every well, including the untreated controls. This stock solution was made by dissolving 5 milligram of MTT in 1 mL of PBS. A 37 °C environment with 5% carbon dioxide was used to incubate the 96-well plates for four hours. After incubation, 85 µL of the medium was removed from each well, and then 50 µL of dimethysulfoxide was added, followed by thorough mixing with a pipette. The wells were then incubated for an additional 10 min at 37 °C. The number of viable cells was calculated using the formula [(ODt/ODc)] × 100%, where ODt is the mean optical density of the wells treated with the tested sample and ODc is the mean optical density of the untreated cells. Optical density was measured at 590 nm using a microplate reader (SunRise, TECAN, Inc., RedwoodCity, CA94063, USA). The survival curve of the tumor cell line following BHCM treatment was generated by plotting the relationship between the number of residual cells and the concentration of the chemical. Using GraphPad Prism software version 10 for Windows (San Diego, CA, USA), dose- response curves for each concentration were created to determine IC_50_ (the 50% inhibitory concentration), which indicates the concentration required to produce harmful effects in 50% of the viable cells [57].

#### 3.3.2. Human Lung Fibroblast Normal Cells (WI-38) 

Propagation of Cell Lines: During the cell culture process, Dulbecco’s Modified Eagle’s Medium (DMEM) was supplemented with 1% L-glutamine, 50 µg/mL gentamicin, HEPES buffer, and 10% heat-inactivated fetal bovine serum. The cells were maintained in a humidified environment with 5% carbon dioxide at 37 °C and were subcultured twice a week.

Viability Assay for Cytotoxicity Evaluation: To conduct the cytotoxicity assay [56], 1 × 10^4^ cells were seeded per well in 100 µL of growth media in 96-well plates. After a 24-h incubation period, fresh medium containing varying concentrations of the test sample was added. Confluent cell monolayers were transferred into flat-bottomed 96- well microtiter plates (Falcon, Newark City, NJ, USA) using a multichannel pipette. The chemical compound being tested (BHCM) was then introduced in repeated two-fold dilutions. The microtiter plates were incubated for 24 h at 37 °C in a humidified incubator with 5% carbon dioxide. Three wells were used for each concentration of the test sample. Control cells were grown with or without dimethysulfoxide, but without the test sample. It was confirmed that the small amount of dimethysulfoxide (maximum 0.1%) present in the wells did not affect the experiment. A colorimetric method was employed to assess the viable cell yield after the incubation period. The MTT assay was employed to quantify the number of viable cells after a 24-h incubation period. In summary, 100 µL of fresh phenol red-free RPMI 1640 culture medium was added to replace the existing medium in the 96-well plate. Following this, each well, including the untreated controls, received 10 µL of a 12 mM MTT stock solution (prepared by dissolving 5 mg of MTT in 1mL of PBS). The 96-well plates were then incubated for four hours at 37 °C in a 5% carbon dioxide environment. After incubation, 85 µL of the medium was removed from each well, and 50 µL of dimethysulfoxide was added. The contents of the wells were mixed thoroughly using a pipette and incubated for an additional 10 min at 37 °C. As stated in Section 3.3.2, the viability percentage, survival curve and 50% inhibitory concentration (IC_50_) were obtained.

#### 3.3.3. Molecular Docking

A simulation method known as molecular docking is used to determine the best place for a ligand to connect with an active site on a target [57,58]. This method entails locating the binding site’s three-dimensional coordinate space in the target and calculating the binding affinity of the molecule’s resulting orientation inside the binding site, which creates the complex. The compounds’ chemical structures were correctly sketched first using ChemDraw Professional Version 16.0 software. The next step was to minimize energy using Chem3D (v.16.0) and the MM2 and MMFF94 tools. Second, the receptors loaded from the protein data bank PDB website: https://www.rcsb.org/ (accessed on 8 May 2024), which are the crystal structures of the Epidermal Growth Factor Receptor (EGFR) protein (PDB code: 5CAO). Compound–target protein interactions were docked using Vina in PyR_X_–Python Prescription 0.8 [59,60]. Using the PyRx software’s Autodock button, the protein and ligand were easily prepared (e.g., by adding hydrogen atoms, removing water molecules, or assigning partial charges), where we decided to make a macromolecule or ligand. Table 2 gives the docking parameters, including exhaustiveness and the details of the grid box employed. Finally, the binding mode and affinity of the compound-5CAO protein were visualized through the use of the PyMol program [61]. The best binding pose was determined based on binding affinity (highest docking score).

## 4. Conclusions

Bis(2-hydroxy-3-carboxyphenyl)methane (BHCM) was synthesized from formaldehyde, sulfuric acid, and salicylic acid. FT-IR, UV–Vis, magnetism, and temperature measurement are effective methods for identifying the structures and compositions of the Ni–BHCM complex. The energy gap values of the present compounds lie in the range of semiconductors, and thus, they could be used as potential materials for harvesting solar radiation in solar cell applications. Complexation of BHCM ligand with Ni(II) decreases the optical band gap (E_g_) of free ligand. It is found that there is a difference in the refractive index (n) values upon chelation. The optical conductivity of the samples can vary/increase with frequency or photon energy. The penetration depth changes with photon energy where the penetration of light through the ligand is greater than the complex. With an IC_50_ of 144.8 ± 10.8 and 361.9 ± 17.8 µM for lung carcinoma and normal cells, respectively, the BHCM exhibited reasonable impacts and can inhibit 50% of cell proliferation. The outcome has the potential to be welcomed, especially because the BHCM structure resembles a naturally occurring and non-toxic salicylic acid. The selected compounds are potent inhibitors of the target proteins, particularly the nickel complex, according to the virtual screening (molecular docking study), and they may one day be employed as prospective medications to treat lung cancer.

## Figures and Tables

**Table 1:** Binding scores of BHCM and Ni–BHCM with 5CAO receptor protein.

Compound	Receptor	Binding Affinity (kcal/mol)	Interacting Amino Acids
L: Bis(2-hydroxy-3-carboxyphenyl)methane (BHCM)	6CAO (implicated in the pathophysiology of lung cancer)	−7.8	Phe-997 and Asp-1012
Complex: (Ni–BHCM)		−10.3	Glu-1015, Lys-846 and Asp-1014

**Table 2:** Docking parameters for BHCM and Ni–BHCM.

Compound	BHCM			Ni–BHCM		
	x	y	z	x	y	z
Center coordinates of the grid box	−60.3605	−7.1502	−22.3618	−60.3605	−7.1502	−25.2943
Dimensions of the grid box (Ǻ)	51.453	65.0735	67.1262	51.4252	65.0735	61.2672
Exhaustiveness	20			20		

## Data Availability

The original contributions presented in the study are included in the article/Supplementary Materials, further inquiries can be directed to the corresponding authors.

## Supplementary Materials

The following supporting information can be downloaded at: https://www.mdpi.com/article/10.3390/molecules29174239/s1, Figure S1. Mass spectrum of BHCM. Figure S2. Proton NMR spectrum of the BHCM in (i) DMSO d_6_ and (ii) DMSO d_6_ + D_2_O. Figure S3. Electronic spectrum of BHCM. Figure S4. XRD graph of BHCM. Figure S5. Variation of optical parameters for BHCM ligand (a) transmittance, T, (b) band gap energy, E_g_, (c) refractive index, n (d) optical conductivity, σ_opt_ and (e) penetration depth, W. Table S1. Substantial IR Assignments of the Ligand and Ni(II) Complex. Table S2: BHCM and Ni(II) Complex XRD Data.

## References

[1] Aglan H.A., Ahmed H.H., El-Toumy S.A., Mahmoud N.S. (2017). Gallic acid against hepatocellular carcinoma: An integrated scheme of the potential mechanisms of action from in vivo study. Tumor Biol..

[2] Li C., Qiu Y., Zhang Y. (2022). Research progress on therapeutic targeting of cancer-associated fibroblasts to tackle treatment-resistant NSCLC. Pharmaceuticals.

[3] Gao Y., Wang G., Wang X., Yang Y., Niu X. (2020). Structure-Activity relationship of MDSA and its derivatives against Staphylococcus aureus Ser/Thr phosphatase Stp1. Comput. Biol. Chem..

[4] Takashi H., Takaaki H., Hyozo S., Alan P.M., Kaipenchery A.K., Simon G.B., Kata M.M., Tatjana S., Nazar S.A.E., Hong-Sik H. (2000). Molecular design of lipophilic disalicylic acid compounds with varying spacers for selective lead(II) extraction. Talanta.

[5] Shrestha S., Bhattarai B.R., Chang K.J., Leea K.-H., Cho H. (2007). Methylenedisalicylic acid derivatives: New PTP1B inhibitors that confer resistance to diet-induced obesity. Bioorganic Med. Chem. Lett..

[6] Trevin S., Bedioui F., Gomez M.G., Charreton C.B. (1997). Electro polymerized nickel Micro cyclic complex-base films design and elctro-catalytic application. J. Mater. Chem..

[7] Parulerkar M.H., Bhatt H.A., Potnis S.P. (1972). New intermediates for plastics and coatings: 1. preparation and characterization of methylene-di-salicylic-acid. J. Ind. Chem. Soc..

[8] Clemmensen E., Heitman A.H.C. (1911). Methylenedisalicylic acid and its reaction with bromine and iodine. J. Am. Chem. Soc..

[9] Sivapullaiah P.V., Soundararajan S. (1976). Methylene di salicylates of rare-earths. J. Indian Inst. Sci..

[10] Patel R.P., Karampurwala A.M., Shah J.R. (1980). Physicochemical studies on square planar Co^2+^, Ni^2+^ and Cu^2+^ chelate polymers. Die Angew. Makromol. Chem. Appl. Macromol. Chem. Phys..

[11] Du M., Zhang Z.H., Guo W., Fu X.J. (2009). Multi-Component Hydrogen-Bonding Assembly of a Pharmaceutical Agent Pamoic Acid with Piperazine or 4,4′-Bipyridyl: A Channel Hydrated Salt with Multiple-Helical Motifs vs a Bimolecular Cocrystal. Cryst. Growth Des..

[12] Wang S., Yun R., Peng Y., Zhang Q., Lu J., Dou J., Bai J., Li D., Wang D. (2012). A Series of Four-Connected Entangled Metal–Organic Frameworks Assembled from Pamoic Acid and Pyridine-Containing Ligands: Interpenetrating, Self-Penetrating, and Supramolecular Isomerism. Cryst. Growth Des..

[13] Wahl H., Haynes D.A., le Roex T. (2012). Porous salts based on the pamoate ion. Chem. Commun..

[14] Wahl H., Haynes D.A., le Roex T. (2011). Solvate formation in lutidinium pamoate salts: A systematic study. CrystEngComm.

[15] Du M., Li C.P., Zhao X.J., Yu Q. (2007). Interplay of coordinative and supramolecular interactions in engineering unusual crystalline architectures of low-dimensional metal–pamoate complexes under co-ligand intervention. CrystEngComm.

[16] Shi X.M., Li M.X., He X., Liu H.-J., Shao M. (2010). Crystal structures and properties of four coordination polymers constructed from flexible pamoic acid. Polyhedron.

[17] Zhang L.N., Sun X.L., Du C.X., Hou H.W. (2014). Structural diversity and fluorescent properties of new metal–organic frameworks constructed from pamoic acid and different N-donor ligands. Polyhedron.

[18] Tang Y.Z., Xiong J.B., Tan Y.H., Wang Y., Deng Y.P., Xu Q., Wen H.R. (2014). Solvothermal syntheses, crystal structures and photoluminescent properties of four coordination polymers with pamoic acid and pyridine mixed ligands. Inorganica Chim. Acta.

[19] Rocha L.D., Monteiro M.C., Anderson J.T. (2012). Anticancer properties of hydroxycinnamic acids—A Review. Cancer Clin. Oncol..

[20] Tanaka T., Tanaka M. (2011). Potential cancer chemopreventive activity of protocatechuic acid. J. Exp. Clin. Med..

[21] Gomes C.A., Cruz T.G., Andrade J.L., Milhazes N., Borges F., Marques M.P.M. (2003). Anticancer activity of phenolic acids of natural or synthetic origin:  A structure–activity study. J. Med. Chem..

[22] Ghasemzadeh A., Jaafar H., Karimi E., Ghasemzadeh A., Jaafar H.Z.E., Karimi E. (2012). Involvement of salicylic acid on antioxidant and anticancer properties, anthocyanin production anthocyanin production and chalcone synthase activity in ginger (Zingiber officinale Roscoe) varieties. Int. J. Mol. Sci..

[23] Bonta R.K. (2020). Dietary phenolic acids and flavonoids as potential anti-cancer agents: Current state of the art and future perspectives. Anti-Cancer Agents Med. Chem..

[24] Calderón-Jiménez B., Montoro-Bustos A.R., Pereira-Reyes R., Paniagua S.A., Vega-Baudrit J.R. (2022). Novel pathway for the sonochemical synthesis of silver nanoparticles with near-spherical shape and high stability in aqueous media. Sci. Rep..

[25] Joseyphus R.S., Nair M.S. (2010). Synthesis, characterization and biological studies of some Co(II), Ni(II) and Cu(II) complexes derived from indole-3-carboxaldehyde and glycylglycine, as Schiff base ligand. Arab. J. Chem..

[26] Rashad M.M., Hassan A.M., Nassar A.M., Ibrahim N.M., Mourtada A. (2014). Anew nano-structured Ni(II) Schiff base complex: Synthesis, characterization, optical band gaps and biological activity. Appl. Phys. A.

[27] Patterson A. (1939). The Scherrer formula for X-ray particle size determination. Phys. Rev..

[28] Sivagami M., Asharani I.V. (2022). Phyto-mediated Ni/NiO NPs and their catalytic applications-a short review. Inorg. Chem. Commun..

[29] Kaya Y., Gençkal H.M., Irez G. (2010). Uv-Vis Spectra and Fluorescence Properties of Two Iminooxime Ligands and Their Metal Complexes: Optical Band Gaps. Gazi Univ. J. Sci..

[30] Kaya I., Koyuncu S., Şenol D. (2006). Conductivity and band gap of oligo-2-[(4-chlorophenyl) imino methylene] phenol and its oligomer–metal complexes. Mater. Lett..

[31] Karipcin F., Dede B., Caglar Y., Hur D., Ilican S., Caglar M., Sahin Y. (2007). A new dioxime ligand and its trinuclear copper(II) complex: Synthesis, characterization and optical properties. Opt. Commun..

[32] Turan N., Gündüz B., Körkoca H., Adigüzel R., Çolak N., Buldurun K. (2014). Study of structure and spectral characteristics of the zinc(II) and copper(II) complexes with 5,5-dimethyl-2-(2-(3-nitrophenyl)hydrazono)cyclohexane-1,3-dione and their effects on optical properties and the developing of the energy band gap and investigation of antibacterial activity. J. Mex. Chem. Soc..

[33] Gittleman J.I., Sichel E.K., Arie Y. (1979). Composite semiconductors: Selective absorbers of solar energy. Sol. Energy Mater..

[34] Belmokhtar A., Yahiaoui A., Hachemaoui A.I., Abdelghani B., Sahli N., Belbachir M. (2011). A NovelPoly{(2,5-diylfuran)(benzylidene)}:Anew synthetic approach and electronic properties. Int. Sch. Res. Not..

[35] Yakuphanoglu F., Erten H. (2005). Refractive index dispersion and analysis of the optical constants of an ionomer thin film. Opt. Appl..

[36] Paul T.C., Podder J. (2019). Synthesis and characterization of Zn-incorporated TiO_2_ thin flms: Impact of crystallite size on X-ray line broadening and bandgap tuning. Appl. Phys. A.

[37] Ahmed A.H. (2020). N,N′-bis[2-hydroxynaphthylidene]/[2-methoxybenzylidene]amino]oxamides and their divalent manganese complexes: Isolation, spectral characterization, morphology, antibacterial and cytotoxicity against leukemia cells. Open Chem..

[38] Kavitha P., Reddy K.L. (2016). Synthesis, spectral characterization, morphology, biological activity and DNA cleavage studies of metal complexes with chromone Schiff base. Arab. J. Chem..

[39] Baxter G.J., Graham A.B., Lawrence J.R., Wiles D., Paterson J.R. (2001). Salicylic acid in soups prepared from organically and non-organically grown vegetables. Eur. J. Nutr..

[40] Wang W., Hong L., Shen Z., Zheng M., Meng H., Ye T., Lin Z., Chen L., Guo Y., He E. (2024). Molecular insights into the anti-spoilage effect of salicylic acid in Favorita potato processing. Food Chem..

[41] Das A.P., Mathur P., Agarwal S.M. (2024). Machine learning, molecular docking and dynamics-based computational identification of potential inhibitors against lung cancer. ACS Omega.

[42] Wang C., Wang X., Wang X., Tian B., Zhang S., Wang T., Ma Y., Fan Y. (2014). Design, synthesis and biological evaluation of potent epidermal growth factor receptor tyrosine kinase (EGFR-TK) inhibitors against resistance mutation for lung cancer treatment. Bioorganic Chem..

[43] Jain A.N. (2003). Surflex: Fully automatic flexible molecular docking using a molecular similarity-based search engine. J. Med. Chem..

[44] Ahmed A.H., Thabet M.S. (2011). Metallo-hydrazone complexes immobilized in zeoliteY: Synthesis, identification and acid violet-1 degredation. J. Mol. Struct..

[45] Drago R.S. (2012). Physical Methods in Inorganic Chemistry PB.

[46] Lewetegn K., Birhanu H., Liu Y., Taddesse P. (2025). Synthesis of Zn_0.87_(Fe, Al)_0.065_O—MO (MO = NiO, Fe_2_O_3_, CuO, and CoO) nanocomposites: Structural, vibrational, optical and antibacterial studies. J. Mol. Struct..

[47] Ahmed A.H., Hassan A.M., Gumaa H.A., Mohamed B.H., Eraky A.M. (2017). Physicochemical studies on some selected oxaloyldihydrazones and their novel palladium(II) complexes along with using oxaloyldihydrazones as corrosion resistants. Inorg. Nano-Met. Chem..

[48] Ahmed A.H., Hassan A.M., Gumaa H.A., Mohamed B.H., Eraky A.M., Omran A.A. (2019). Copper(II)-oxaloyldihydrazone complexes: Physico-chemicalstudies; energy band gap and inhibition evaluation of free oxaloyldihydrazones toward the corrosion of copper metal in acidic medium. Arab. J. Chem..

[49] Haque R.A., Iqbal M.A., Khadeer M.B. (2012). Design, synthesis and structural studies of meta-xylyl linked bis-benzimidazolium salts: Potential anticancer agents against human colon cancer. Chem. Cent. J..

[50] Lal R.A., Basumatary D., Arjun K.D., Kumar A. (2007). Synthesis and spectral characterization of zinc(II), copper(II), nickel(II) and manganese(II) complexes derived from bis(2-hydroxy-1-naphthaldehyde) malonoyldihydrazone. Transit. Met. Chem..

[51] Ferraro J.R. (1971). Low Frequency Vibration of Inorganic and Coordination Compounds.

[52] Nicholls D. (1974). Complexes and First-Row Transition Elements, London and Basingstoke.

[53] Ahmed A.H., Moustafa M.G. (2020). Spectroscopic, morphology and electrical conductivity studies on Co(II), Ni(II), Cu(II) and Mn(II)- oxaloyldihydrazone complexes. J. Saudi Chem. Soc..

[54] Lee J.D. (1996). Consize Inorganic Chemistry.

[55] Abo-Ashour M.F., Eldehna W.M., Nocentini A., Bonardi A., Bua S., Ibrahim H.S., Elaasser M.M., Kryštof V., Jorda R., Gratteri P. (2019). 3-Hydrazinoisatin-based benzenesulfonamides as novel carbonic anhydrase inhibitors endowed with anticancer activity: Synthesis, in vitro biological evaluation and in silico insights. Eur. J. Med. Chem..

[56] Gomha S.M., Riyadh S.M., Mahmmoud E.A., Elaasser M.M. (2015). Synthesis and anticancer activities of thiazoles, 1,3-thiazines, and thiazolidine using chitosan-grafted-poly(vinylpyridine) as basic catalyst. Heterocycles.

[57] Albqmi M., Elkanzi N.A.A., Ali A.M., Abdou A. (2025). Design, Characterization, and DFT exploration of new mononuclear Fe(III) and Co(II) complexes based on Isatin-hydrazone derivative: Anti-inflammatory profiling and molecular docking insights. J. Mol. Struct..

[58] Mert S., Demir Y., Sert Y., Kasımoğulları R., Gülçin İ. (2025). Synthesis, biological evaluation and molecular docking of novel pyrazole derivatives as multitarget acetylcholinesterase and carbonic anhydrase inhibitors. J. Mol. Struct..

[59] Dallakyan S., Olson A.J. (2015). Small-molecule library screening by docking with PyRx. Chem. Biol. Methods Protoc..

[60] Coumar M.S. (2021). Molecular Docking for Computer-Aided Drug Design: Fundamentals, Techniques, Resources and Applications.

[61] Zhang Y., Zhao Z., Li W., Tang Y., Wang S. (2023). Mechanism of Taxanes in the Treatment of Lung Cancer Based on Network Pharmacology and Molecular Docking. Curr. Issues Mol. Biol..

