# Supplementary material for: Characterization and Cytotoxic Assessment of Bis(2-hydroxy-3-carboxyphenyl)methane and Its Nickel(II) Complex
Source: Molecules. 2024 Sep 6;29(17):4239. doi: 10.3390/molecules29174239 (PMC11397195; doi:10.3390/molecules29174239)
Supplement: Supplementary file 1 [file molecules-29-04239-s001.zip › molecules-3165474-supplementary.pdf]

# Supplementary material

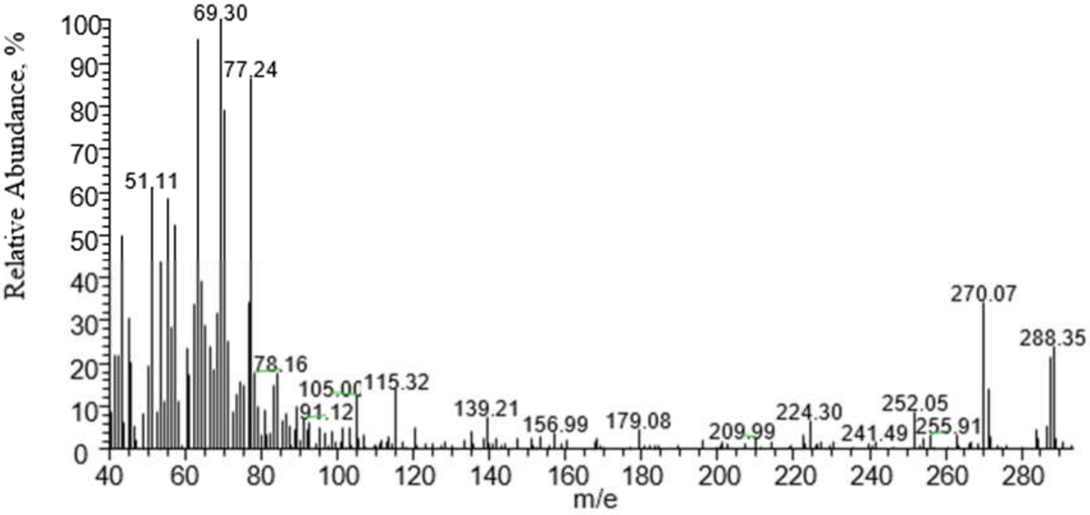


**Figure S1.** Mass spectrum of BHCM.

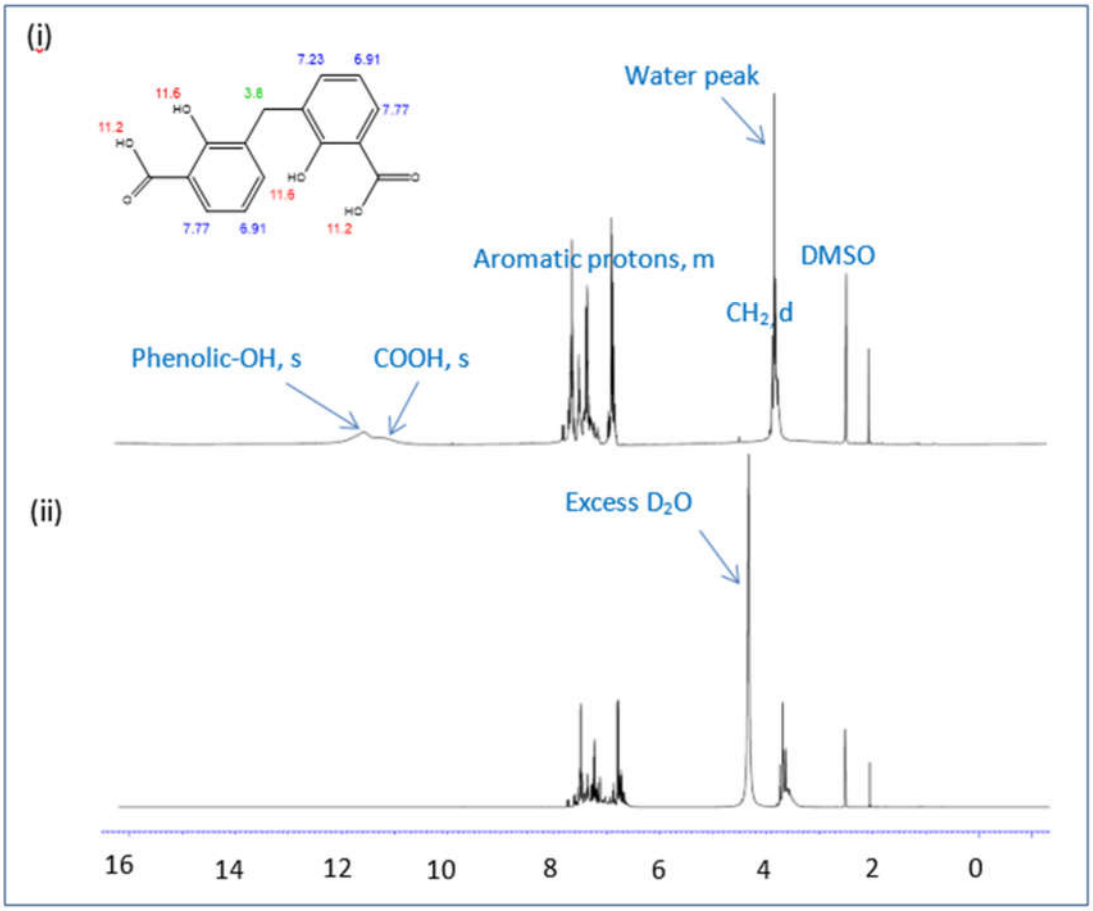


*Note: Numbers on the structure point to $\delta_{ppm}$ acquired by proton NMR analysis*

**Figure S2. P**roton NMR spectrum of the BHCM in (i) DMSO $d_6$ and (ii) DMSO $d_6$ + $D_2O$.

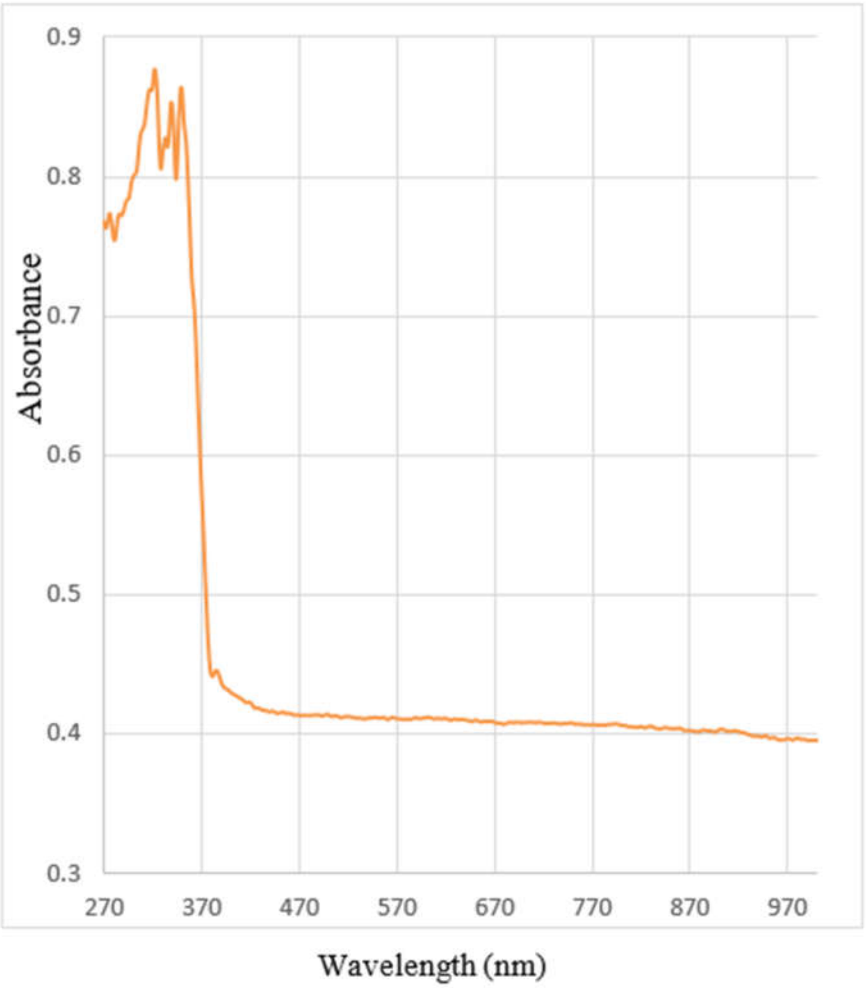


**Figure S3.** Electronic spectrum of BHCM.

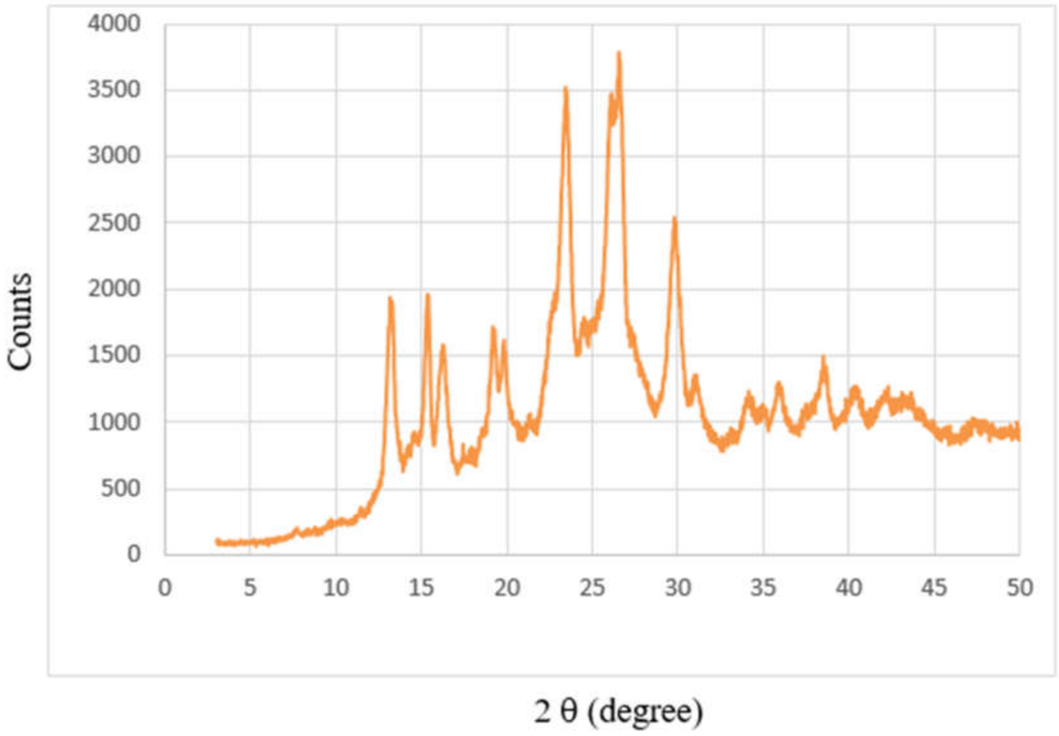


**Figure S4.** XRD graph of BHCM.

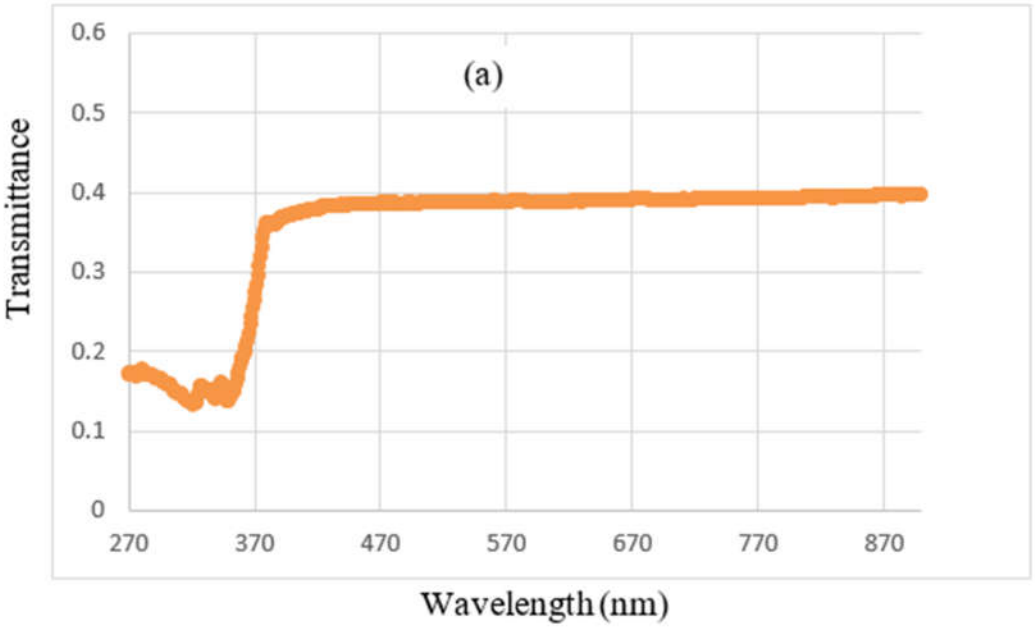
(a)
Transmittance
0.6
0.5
0.4
0.3
0.2
0.1
0
270
370
470
570
670
770
870
Wavelength (nm)

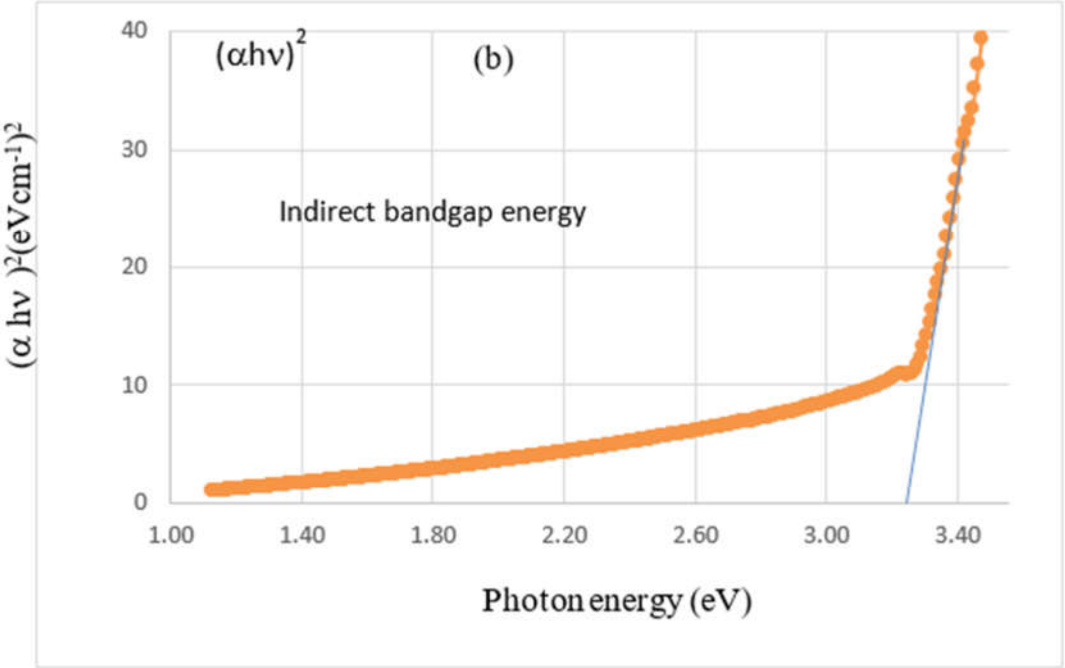
$(\alpha h\nu)^2$
(b)
Indirect bandgap energy
$(\alpha h\nu)^2 (eVcm^{-1})^2$
40
30
20
10
0
1.00
1.40
1.80
2.20
2.60
3.00
3.40
Photon energy (eV)

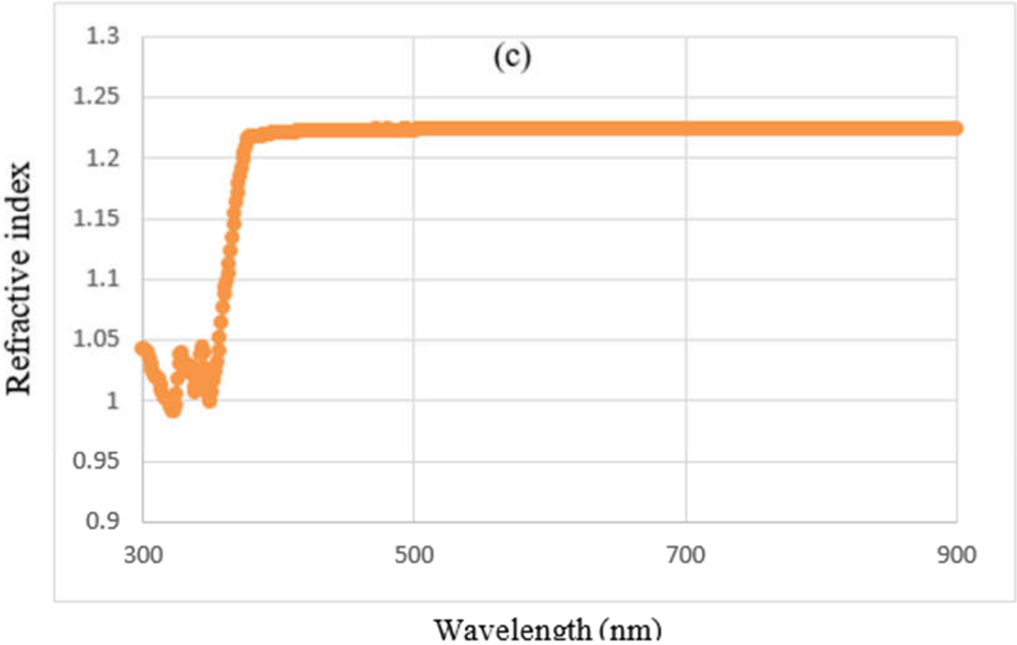
(c)
Refractive index
1.3
1.25
1.2
1.15
1.1
1.05
1
0.95
0.9
300
500
700
900
Wavelength (nm)

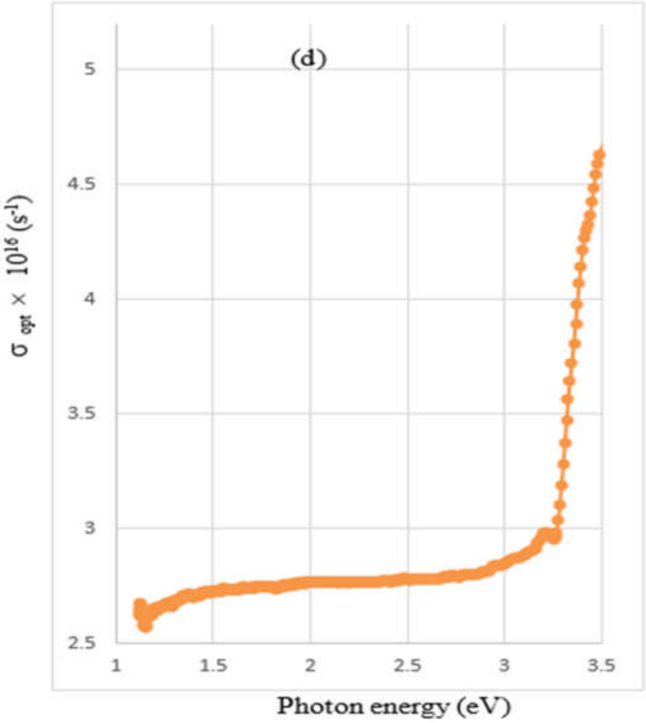
(d)
$\sigma_{opt} \times 10^{16}$ ($s^{-1}$)
5
4.5
4
3.5
3
2.5
1
1.5
2
2.5
3
3.5
Photon energy (eV)

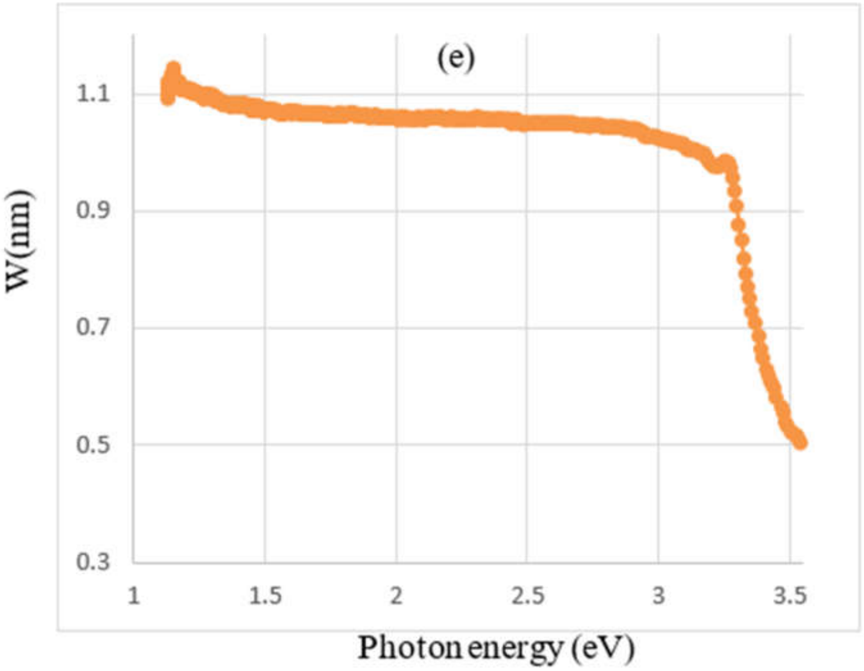


**Figure S5.** Variation of optical parameters for BHCM ligand (a) transmittance, T, (b) band gap energy, $E_g$, (c) refractive index, n (d) optical conductivity, $\sigma_{opt}$ and (e) penetration depth, W.

**Table S1. Substantial IR Assignments of Ligand and Ni(II) Complex.**

| Compound (formula/ molecular weight) | υ(OH) phenolic (H-bond) | δ(OH) phenolic | υ(C-O) phenolic | υ(OH) carboxylic (H-bond) | υ(C=O) carboxylic (L) / carboxylate (complex) | υ(Ni-O) phenolic/carboxylate | $\mu_{eff}$ (B.M) | Symmetry |
|---|---|---|---|---|---|---|---|---|
| Ligand: BHCM ($C_{15}H_{12}O_6$ / 288.26) | ≈3150 | 1200 | 1281 | 2390-2650 | 1647 | - | - | - |
| Complex: Ni-BHCM ($C_{15}H_{38}O_{21}Ni_2$)/ 671.83) | - | - | 1245 | - | 1560 | 544, 651 | 5.35 | Oh |

**Table S2: BHCM and Ni(II) Complex XRD Data.**

| Compound | XRD data | | | |
|---|---|---|---|---|
| | Angle (d-value) | θ° | β (rad) | D (nm) |
| BHCM | 13.206° (6.69913 Å), 15.324° (5.77747 Å), 16.239°(5.45398 Å), 19.169° (4.62643 Å), 19.797° (4.48108 Å), 22.694° (3.91519 Å), 23.424° (3.79470 Å), 26.521° (3.35814 Å), 29.789° (2.99686 Å), 30.990° (2.88335 Å), 34.075° (2.62905 Å), 35.858° (2.50227 Å), 38.488° (2.33714 Å), 40.300° (2.23612 Å), 42.072° (2.14595 Å) | 23.47 | 0.011 | 14.4 |
| Ni-BHCM | 7.522° (11.74372 Å) | - | - | - |
